# Suppression of β3-integrin in mice triggers a neuropilin-1-dependent change in focal adhesion remodelling that can be targeted to block pathological angiogenesis

**DOI:** 10.1242/dmm.019927

**Published:** 2015-09-01

**Authors:** Tim S. Ellison, Samuel J. Atkinson, Veronica Steri, Benjamin M. Kirkup, Michael E. J. Preedy, Robert T. Johnson, Christiana Ruhrberg, Dylan R. Edwards, Jochen G. Schneider, Katherine Weilbaecher, Stephen D. Robinson

**Affiliations:** 1 School of Biological Sciences, University of East Anglia, Norwich Research Park, Norwich, NR4 7TJ, UK; 2 Institute of Ophthalmology, University College London, London, EC1V 9EL, UK; 3 Luxembourg Center for Systems Biomedicine (LCSB), University of Luxembourg, Luxembourg & Saarland University Medical Center, Internal Medicine II, L-4362 Homburg, Germany; 4 Department of Internal Medicine, Division of Molecular Oncology, Washington University in St Louis, St Louis, MO 63110, USA

**Keywords:** Integrin, Neuropilin-1, Angiogenesis, Tumour, Focal adhesion

## Abstract

Anti-angiogenic treatments against αvβ3-integrin fail to block tumour growth in the long term, which suggests that the tumour vasculature escapes from angiogenesis inhibition through αvβ3-integrin-independent mechanisms. Here, we show that suppression of β3-integrin in mice leads to the activation of a neuropilin-1 (NRP1)-dependent cell migration pathway in endothelial cells via a mechanism that depends on NRP1's mobilisation away from mature focal adhesions following VEGF-stimulation. The simultaneous genetic targeting of both molecules significantly impairs paxillin-1 activation and focal adhesion remodelling in endothelial cells, and therefore inhibits tumour angiogenesis and the growth of already established tumours. These findings provide a firm foundation for testing drugs against these molecules in combination to treat patients with advanced cancers.

## Abstract (precis)

**Summary:** Targeting both β3-integrin and neuropilin-1 prevents anti-angiogenic treatment escape.

## INTRODUCTION

Angiogenesis, the formation of new blood vessels from pre-existing vasculature, is essential to support both primary and metastatic tumour growth (Hanahan and Weinberg, 2011). It occurs when hypoxia causes tumour cells to release growth factors such as vascular endothelial growth factor (VEGF), which stimulate nearby endothelial cells (ECs) to activate appropriate growth factor receptors, e.g. VEGF-receptor-2 (VEGFR2). New blood-vessel formation ensues as ECs proliferate and migrate through the extracellular matrix (ECM) toward the tumour in an integrin-dependent fashion (Robinson and Hodivala-Dilke, 2011). This process of tumour angiogenesis frequently offers a route to metastasis by providing an increased density of highly permeable blood vessels. Thus, anti-angiogenic strategies form a key component of the current cancer-targeting arsenal. Because of its central role in the process, many of the existing anti-angiogenic strategies target the VEGF-VEGFR2 pathway, but these approaches are linked to a plethora of unwanted side effects and the development of treatment resistance (Ebos and Kerbel, 2011).

Integrins are the main ECM adhesion receptors. They sense, integrate and disseminate ECM and growth factor signals to co-ordinate EC responses during angiogenesis (Silva et al., 2008). αvβ3-integrin has emerged as a key anti-angiogenic therapeutic target because it is upregulated in the vasculature of solid tumours, but its expression is low in quiescent vasculature (Brooks et al., 1994). Unlike current FDA-approved anti-angiogenic drugs, αvβ3-integrin antagonists are well-tolerated, likely owing to the fact that αvβ3-integrin expression is restricted to neo-angiogenic vessels (Hariharan et al., 2007). However, the synthetic inhibitors directed against αvβ3-integrin have so far failed to improve overall survival in patients (Marelli et al., 2013). Moreover, although we have shown that suppressing endothelial αvβ3-integrin in the early stages of tumour growth has an inhibitory growth effect, its long-term suppression leads to ‘treatment’ resistance (Steri et al., 2014). We do not yet understand what the mechanisms of resistance are, or how to overcome them. In order to improve therapeutic outcomes when targeting this key molecule, we need to rethink how to best use anti-angiogenic strategies based on αvβ3-integrin antagonism.

A promising approach to overcome resistance to anti-angiogenic treatment is to identify pathways of resistance and to co-target them alongside the original target. Such approaches are emerging in a number of cancer types as viable therapeutic strategies to improve upon existing treatments (Sennino and McDonald, 2012). In 2009, an αvβ3-integrin co-target candidate emerged: the VEGF co-receptor, neuropilin-1 (NRP1) (Robinson et al., 2009). However, we do not yet fully understand how these two molecules cooperate mechanistically to regulate pathological angiogenesis, nor do we know whether their interaction can be manipulated to alter outcomes.

NRP1 is a single-pass transmembrane molecule found, for example, in neurons and ECs. Originally identified as an adhesion molecule (Takagi et al., 1995), attention shifted to its VEGF-dependent role in binding and regulating the signalling and trafficking of VEGFR2 in ECs (Ballmer-Hofer et al., 2011; Herzog et al., 2011). NRP1's short cytoplasmic tail is crucial for the regulation of VEGFR2 trafficking because its SEA motif binds the PDZ domain of synectin, linking the complex to the inward trafficking motor myosin VI (Ballmer-Hofer et al., 2011; Cai and Reed, 1999). Recent work has returned to its original role as an adhesion molecule. Valdembri et al. (2009) showed that NRP1 binds and regulates α5β1-integrin trafficking, again via its cytoplasmic tail, and thus promotes EC spreading on low concentrations of fibronectin (FN), whereas Raimondi et al. (2014) demonstrated an essential role for NRP1 in promoting FN-dependent signalling in ECs. In support of VEGF-independent roles for NRP1 in ECs, Fantin et al. (2014) found that mice with a mutant NRP1 defective in VEGF binding overcome the mid-gestation lethality of full NRP1-knockout mice (Kawasaki et al., 1999). Indeed, there is mounting evidence that links VEGF-independent roles for NRP1 to focal adhesion (FA) function (Raimondi et al., 2014; Seerapu et al., 2013). What we do not yet know is how and when, and in what cell types, NRP1 contributes to the assembly and maintenance of these large and dynamic macromolecular assemblies that link the ECM to the cytoskeleton and through which both mechanical force and regulatory signals are transmitted (Zamir and Geiger, 2001).

**TRANSLATIONAL IMPACT**

**Clinical issue**

αvβ3-integrin has emerged as a key anti-angiogenic target in cancer therapy because it plays a pivotal role in endothelial cell migration and vascular endothelial growth factor (VEGF)-mediated signalling. However, innate and acquired treatment resistance occurs with its blockade, so treatment fails to block tumour growth in the long term. Understanding the molecular mechanisms that contribute to this resistance is necessary for developing new strategies that target αvβ3-integrin to inhibit tumour growth and progression. It is clear that alternative pro-angiogenic pathways become active in the absence of αvβ3-integrin expression, which suggests that these pathways offer routes to resistance to αvβ3-integrin blockade. This study seeks to understand the molecular basis behind how αvβ3-integrin-regulated pathways contribute to anti-angiogenic resistance and to test the hypothesis that blocking αvβ3-integrin in combination with these pathways will block tumour progression.

**Results**

Using heterozygous β3-integrin-deficient mice as a model of αvβ3-integrin blockade, the authors uncovered a neuropilin-1 (NRP1)-regulated endothelial-cell-migration pathway that only becomes active when αvβ3-integrin expression is suppressed. In β3-integrin heterozygous endothelial cells, but not in wild type, NRP1 regulated paxillin-1 phosphorylation, focal adhesion remodelling and cell migration. This newly found role for NRP1 correlates with its mobilisation away from mature focal adhesions in VEGF-induced β3-integrin-depleted cells; this shift in cellular localisation does not occur in wild-type cells. Finally, the authors show that suppressing the endothelial expression of both αvβ3-integrin and NRP1 blocks the progression of already-established tumours.

**Implications and future directions**

This study uncovered an NRP1-dependent migration pathway that only becomes active upon αvβ3-integrin depletion and it showed that this pathway can be targeted to block tumour progression. These results implicate endothelial NRP1 as a potential co-target during αvβ3-integrin-directed therapies to prevent anti-angiogenic treatment resistance.

That NRP1 plays a crucial role in developmental angiogenesis and arteriogenesis is undeniable (Fantin et al., 2014, 2011; Gerhardt et al., 2004; Kawasaki et al., 1999; Lanahan et al., 2013), but its role in pathological angiogenesis is not clear. Although a number of papers allude to it having no role, Fantin et al. (2014) published findings suggesting that the VEGF-binding domain of NRP1 is important for pathological neovascularisation of the retina and angiogenesis-dependent tumour growth. Here, we conclusively show, however, that disrupting NRP1 function by deleting its cytoplasmic tail or by depleting its expression in wild-type mice has no effect on tumour growth or angiogenesis. However, we can sensitise angiogenic responses to NRP1 perturbations by reducing β3-integrin expression in heterozygous β3-integrin-deficient mice. We show that β3-integrin expression is essential for the efficient retention of NRP1 at FAs after VEGF-stimulation. NRP1 can influence paxillin-1 (PXN) activity and FA remodelling, but only if it is relieved from its retention within mature FAs by reducing β3-integrin expression. This sensitisation to NRP1 perturbations that occurs upon suppressing β3-integrin means that we can target both molecules simultaneously to significantly improve inhibition of growth and angiogenesis in both new and established tumours. This finding offers a potential solution to improve anti-angiogenic strategies.

## RESULTS

### Pathological angiogenesis is sensitive to NRP1 disruption in heterozygous β3-integrin-deficient mice

We previously showed that VEGF-mediated angiogenic responses become dependent on NRP1 in β3-integrin-knockout (β3-KO) mice (Robinson et al., 2009). In this model, angiogenesis was significantly blocked only by simultaneously inhibiting both β3-integrin and NRP1, a finding that we would like to translate to the clinic. In the β3-KO model, however, pathological angiogenesis is elevated over the wild type (Reynolds et al., 2002). It has been postulated that this phenotypic response occurs, in part, through the developmental upregulation of VEGFR2 (Reynolds et al., 2004), making it potentially difficult to interpret experimental outcomes that depend on molecular interactions with this growth-factor receptor. We therefore moved our analyses to β3-integrin-heterozygous (β3-HET) mice, hypothesising that this would circumvent developmental changes arising from the complete loss of the protein, whilst at the same time maintaining, at least to a degree, critical interactions between β3-integrin and VEGFR2 (Mahabeleshwar et al., 2007) and/or NRP1 (Robinson et al., 2009).

**Fig. 1.**
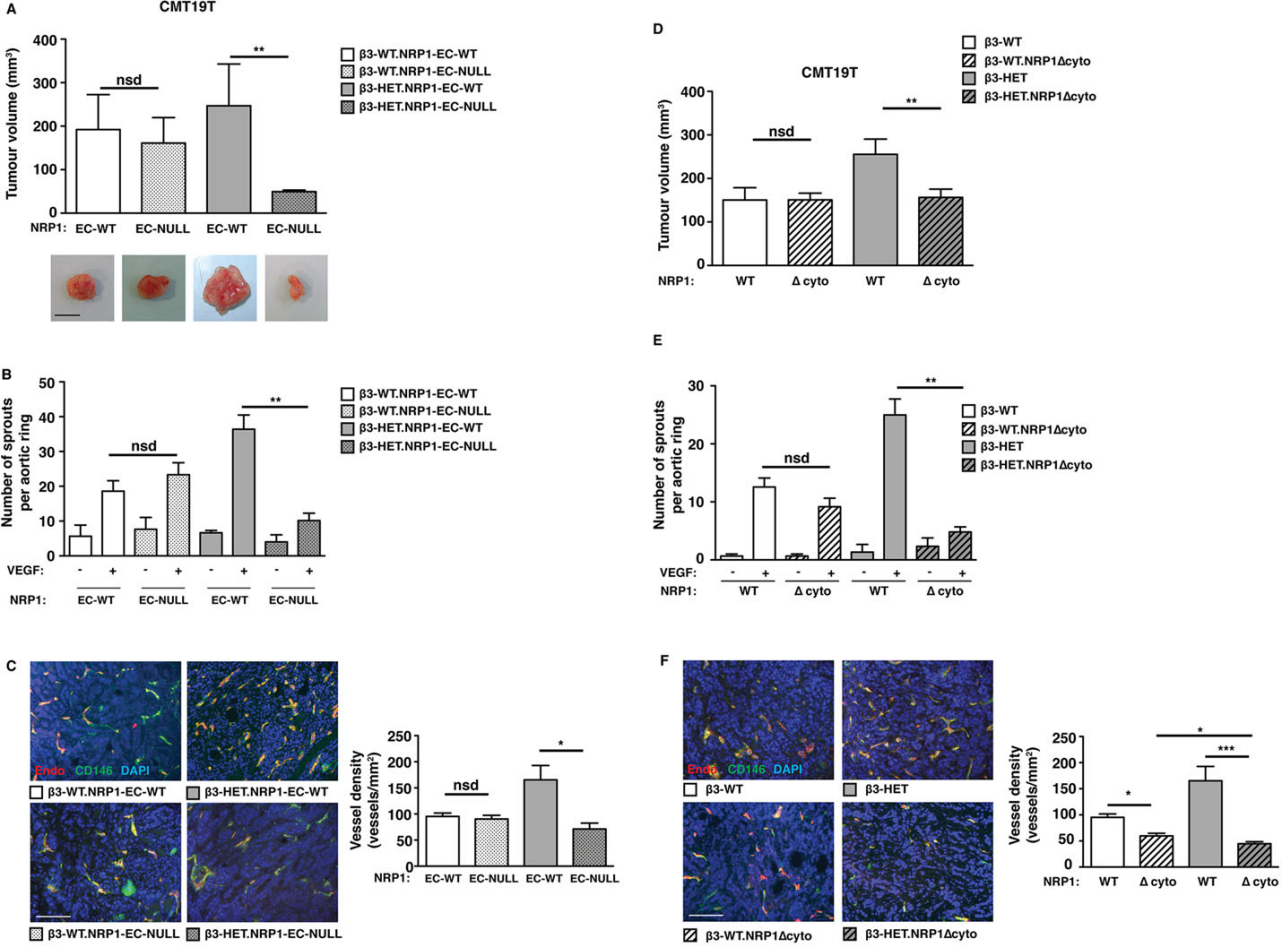
**Tumour growth, tumour angiogenesis and microvessel sprouting in β3-integrin-deficient heterozygous mice are sensitive to NRP1 perturbations.** (A) Tumour growth was measured in animals of the indicated genotypes. Mice were given subcutaneous injections of CMT19T tumour cells. To generate NRP1-EC-KO (EC-null), 21-day slow-release OHT pellets were administered 3 days prior to tumour-cell injection. OHT-treated Cre-negative (NRP-EC-WT) littermates served as controls. Tumour volumes were measured after 12 days of growth (mean+s.e.m. of three independent experiments; *n*≥10 animals per genotype). Representative pictures of tumour macroscopic appearances are shown. Scale bar: 10 mm. (B) Microvessel sprouting of aortic ring explants of the indicated genotypes. NRP1-EC-KO was induced in culture with 1 μM OHT. OHT-treated Cre-negative (EC-NRP-WT) rings served as controls. The bar chart shows the total number of microvessel sprouts per aortic ring after 6 days of VEGF-stimulation (mean+s.e.m. from three independent experiments; *n*≥40 rings per genotype). (C) Blood-vessel density was assessed in tumours of the indicated genotypes by counting the total number of endomucin-positive vessels across tumour sections (mean+s.e.m.; *n*≥10 sections per genotype over three independent experiments). Representative micrographs of immunofluorescence staining for endomucin, an endothelial cell marker (Endo; red) and CD146, a pericyte marker (green) in tumour sections from each genotype are shown. DAPI (blue) was used as a nuclear counterstain. Scale bar: 100 μm. (D) CMT19T tumour growth and angiogenesis were measured in animals of the indicated genotypes. In addition to their β3-integrin genetic status, mice were negative (NRP1 WT) or positive (NRP1 Δcyto) for the loss of NRP1's cytoplasmic tail. Mice were given subcutaneous injections of CMT19T cells and tumour volumes were measured 12 days later. The bar chart shows tumour volumes (mean+s.e.m. of three independent experiments; *n*≥10 animals per genotype). (E) Microvessel sprouting of aortic ring explants of the indicated genotypes. The bar chart shows the total number of microvessel sprouts per aortic ring after 6 days of VEGF-stimulation (mean+s.e.m. from three independent experiments; *n*≥40 rings per genotype). (F) Blood-vessel density was assessed by endomucin (red) and CD146 (green) staining (mean+s.e.m.; *n*≥10 sections per genotype). DAPI was used as a nuclear counterstain (blue). Scale bar: 100 μm. Asterisks indicate statistical significance: *P<0.05; **P<0.01; ***P<0.001; nsd, not significantly different. Unpaired two-tailed *t*-test.

We crossed β3-integrin-wild-type (β3-WT) and β3-HET mice to tamoxifen (OHT)-inducible Pdgfb-iCreER^T2^/NRP1-floxed mice (Claxton et al., 2008; Gu et al., 2003) and examined the effect of an acute EC-specific depletion of NRP1 (EC-NRP1-KO) on subcutaneous allograft tumour growth with both CMT19T cells (Fig. 1A) and B16F0 cells (supplementary material Fig. S1), as well as on aortic ring sprouting (Fig. 1B). Depleting EC-NRP1 expression in this way had no effect on β3-WT responses, but significantly inhibited tumour growth and VEGF-induced microvessel sprouting in β3-HET mice. Tumour angiogenesis was significantly inhibited in β3-HET mice by depleting EC-NRP1, although vessel morphology and pericyte coverage were normal (Fig. 1C). These studies are reminiscent of the changes observed in β3-KO mice, but, importantly, suggest that NRP1 function is already perturbed by subtle changes in β3-integrin expression levels.

To narrow our mechanistic focus further, we crossed β3-WT and β3-HET animals with mice carrying a global deletion of NRP1's cytoplasmic tail (NRP1Δcyto), which is essential for many of its functions (Fantin et al., 2011; Lanahan et al., 2013). CMT19T tumour growth (Fig. 1D) and microvessel sprouting (Fig. 1E) patterns were unaltered in β3-WT mice by the introduction of the NRP1Δcyto mutation, whereas both parameters were inhibited in β3-HET mice. Although the loss of NRP1's cytoplasmic tail had a small effect on tumour angiogenesis in β3-WT mice (Fig. 1F), this did not translate to an overall difference in tumour growth (Fig. 1D). As in EC-NRP1-KO tumours, pericyte coverage of tumour vasculature was not affected by an NRP1 cytoplasmic deletion (Fig. 1F). We conclude from these studies that NRP1's cytoplasmic tail is normally dispensable for pathological angiogenesis, but plays a significant role when insufficient β3-integrin is expressed.

### NRP1-dependent functions of VEGFR2 are normal in β3-integrin-heterozygous endothelial cells

**Fig. 2.**
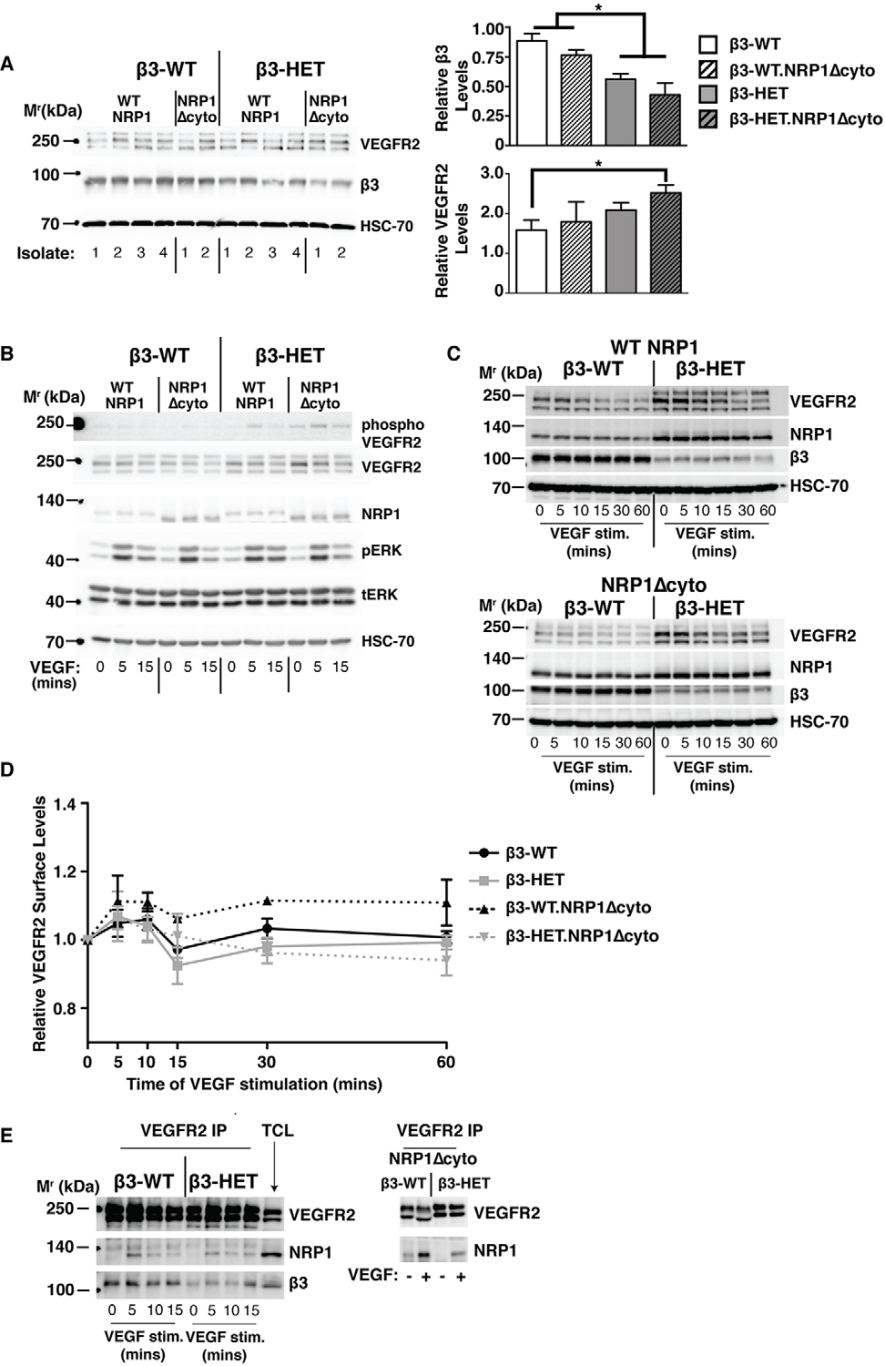
**NRP1-dependent functions of VEGFR-2 are normal in β3-integrin-heterozygous endothelial cells.** (A) Lung microvascular endothelial cells (ECs) were isolated and immortalised (polyoma-middle-T-antigen) from β3-WT and β3-HET mice that were expressing either normal (WT NRP1) or cytoplasmic-tail-deleted (NRP1 Δcyto) NRP1. Multiple EC lysates of each genotype were western blotted (WB) to examine total cellular levels of VEGFR2 and β3-integrin. Bar charts of densitometric analysis of mean (+s.e.m.) changes between the four genotypes are shown to the right. Asterisks indicate statistical significance: *P<0.05. (B-E) Representative of ≥three independent experiments per blotted protein. ECs were seeded overnight on a complex matrix containing gelatin, collagen, fibronectin and vitronectin, and were then stimulated with VEGF over the indicated time courses. (B) ECs were lysed and blotted for levels of phosphorylated (phospho) and total VEGFR2, NRP1, and phospho (pERK) and total (tERK) ERK1/2. (C) To examine protein degradation, the VEGF time course was extended and EC lysates were blotted for levels of total VEGFR2, NRP1 and β3-integrin. (D) Following a VEGF time course, ECs were trypsinised and analysed by flow cytometry for surface levels of VEGFR2. Median fluorescence intensity was measured after forward versus side scatter data were tightly gated around, and normalised to, an isotype control. The graph shows the relative surface level of VEGFR2 (means±s.e.m.) relative to the 0 (non-stimulated) time point. (E) ECs were stimulated with VEGF for the indicated amounts of time and then lysed and immunoprecipitated for VEGFR2 (VEGFR2 IP), before being blotted for NRP1 association. A total cell lysate (TCL) is shown for comparison. (A-E) HSC-70 served as a loading control. Data are representative of three independent experiments.

Given that NRP1's cytoplasmic tail is important for the regulation of VEGFR2 signalling and trafficking (Ballmer-Hofer et al., 2011; Herzog et al., 2011), we wanted to investigate whether the NRP1Δcyto mutation differentially affects VEGFR2 function in β3-WT versus β3-HET lung microvascular ECs. We employed polyoma-middle-T-antigen immortalised ECs, isolated from the mutant mice described above, because we and others have shown that they present good models to study angiogenesis (Ni et al., 2014; Robinson et al., 2009; Steri et al., 2014; Tavora et al., 2014). We first compared total VEGFR2 levels in the four genotypes (β3-WT, β3-HET, β3-WT;NRP1Δcyto and β3-HET;NRP1Δcyto). Unlike β3-KO ECs, we noted only a small trend of increased VEGFR2 levels in β3-HET and β3-HET;NRP1Δcyto ECs (Fig. 2A).

To explore potential changes in VEGFR2-dependent signalling, we seeded ECs on a complex matrix containing gelatin, collagen I (COLI), FN and vitronectin (VN) to preserve the known VN-dependent β3-VEGFR2 interaction (Soldi et al., 1999), stimulated with VEGF, and immunoblotted for changes in total and phosphorylated levels of VEGFR2 and its downstream targets ERK1/2 (Koch et al., 2011) (Fig. 2B). Although VEGF-induced VEGFR2 phosphorylation was slightly elevated in β3-HET ECs, VEGFR2 phosphorylation was not significantly changed by the introduction of the NRP1Δcyto mutation in either β3-WT or β3-HET ECs, suggesting that VEGFR2 signalling is NRP1-independent in these microvascular ECs despite differences in β3-integrin expression. In contrast, VEGF-induced ERK1/2 phosphorylation was sensitive to an NRP1 cytoplasmic deletion in β3-HET ECs but not β3-WT ECs (Fig. 2B).

We observed no overt changes in the pattern of total cellular VEGFR2 expression over time after VEGF stimulation when comparing the four genotypes to one another (Fig. 2C). In each case, VEGFR2 expression levels dropped over the time course of stimulation. This is suggestive of the protein being degraded over time in all four genotypes and is congruent with prior reports (Ewan et al., 2006; Reynolds et al., 2009; Reynolds and Hodivala-Dilke, 2006).

Taken together, these signalling studies pointed to a VEGFR2-independent role for NRP1 in mediating VEGF-induced responses in β3-HET ECs. However, as previously mentioned, when compared to their β3-WT counterparts, total VEGFR2 levels were slightly elevated in both β3-HET and β3-HET;NRP1Δcyto ECs. Moreover, NRP1 expression was substantially increased in both β3-WT;NRP1Δcyto and β3-HET;NRP1Δcyto ECs (Fig. 2A,B). We therefore decided to examine VEGFR2 behaviour in greater detail, including its direct interactions with NRP1.

We measured VEGFR2 surface levels via flow-cytometry over a VEGF-stimulated time course (Fig. 2D). However, surface levels were similar between the four genotypes, and were as predicted from previously published studies (Lampugnani et al., 2006; Reynolds et al., 2009). Because VEGF-induced interactions between VEGFR2 and NRP1 are elevated in β3-KO ECs (Robinson et al., 2009), we next examined this interaction in β3-HET ECs by co-immunoprecipitation, but observed no alterations compared to β3-WT ECs (Fig. 2E). In contrast to previously published studies (Prahst et al., 2008), we saw that the VEGF-induced association between the two molecules was preserved in both β3-WT;NRP1Δcyto and β3-HET;NRP1Δcyto ECs (Fig. 2E). We also examined by immunoblotting other known NRP1-dependent signalling pathways, including the activation of focal adhesion kinase (FAK) (Herzog et al., 2011) and p130Cas (Evans et al., 2011), but saw no changes that might explain why β3-HET mice are sensitive to NRP1 perturbation, whereas β3-WT mice are not (supplementary material Fig. S2); both pathways were inhibited in both genotypes by the introduction of the NRP1Δcyto mutation.

Although these analyses did not rule out subtle differences between the four genotypes in VEGFR2 trafficking, they did allow us to draw an important conclusion: the differential sensitivity of β3-WT and β3-HET animals to NRP1 disruption does not seem to arise from overt changes in VEGFR2 function.

### The VEGF-induced migration in β3-integrin-heterozygous microvascular endothelial cells depends on the cytoplasmic tail of NRP1

**Fig. 3.**
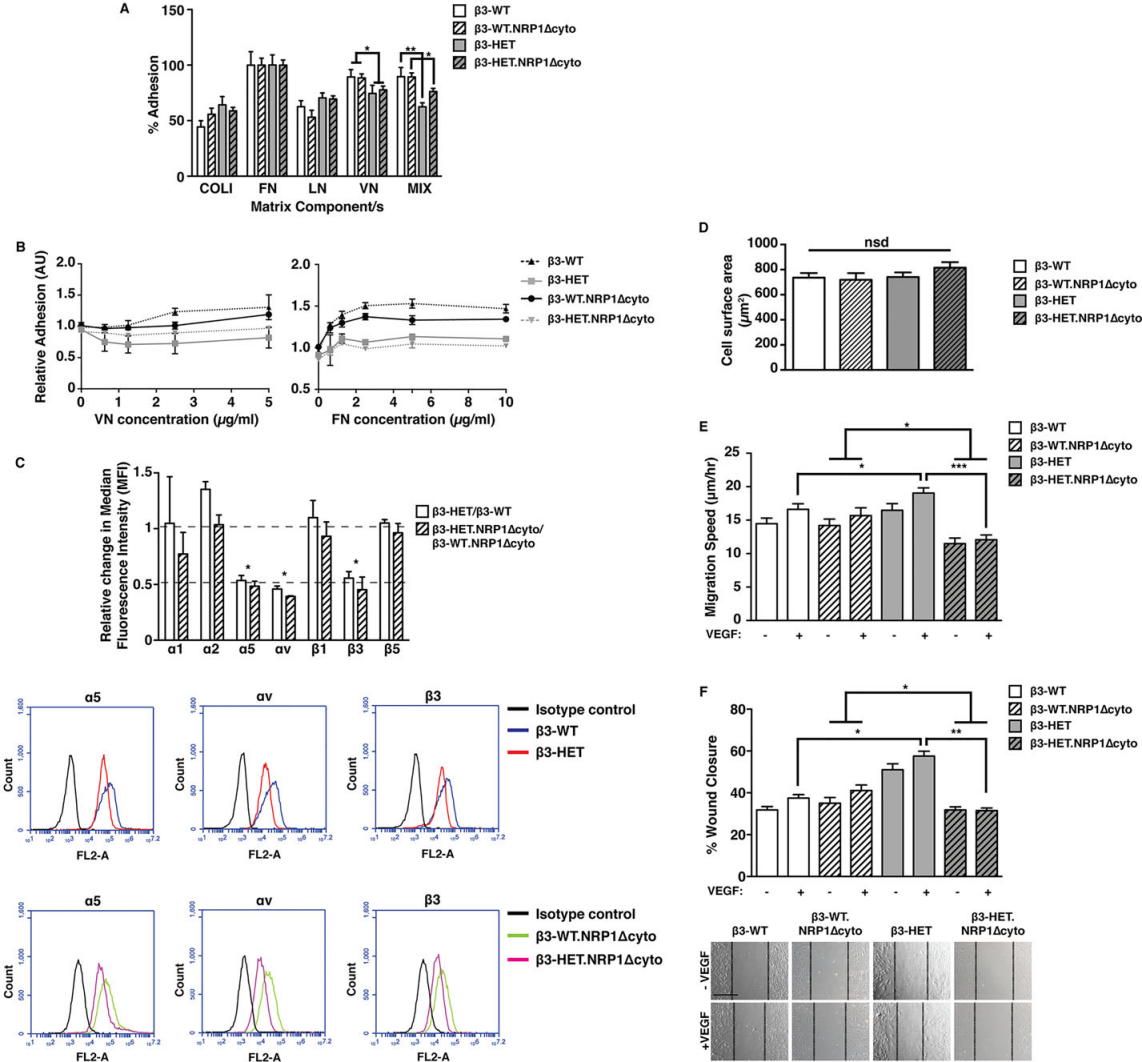
**VEGF-induced migration in β3-integrin-heterozygous endothelial cells is dependent on NRP1.** (A) ECs isolated from animals of the four indicated genotypes were seeded on collagen type I (COLI), fibronectin (FN), laminin (LN), vitronectin (VN), or a complex mixture of COLI, FN, VN and gelatin (MIX) for 90 min. Unattached cells were gently washed off, and the remaining cells were fixed and stained. Dye was extracted and measured spectophotometrically. The bar chart shows the percentage of cell adhesion to each component relative to FN (means+s.e.m. from three independent experiments). (B) ECs of the indicated genotypes were plated on increasing concentrations of FN or VN. After 90 min, plates were vigorously washed and remaining cells were fixed and stained. Dye was extracted and measured spectophotometrically. The graph shows the mean (±s.e.m. from ≥two independent experiments) number of cells that remained attached to the plate after the procedure. (C) ECs of the indicated genotypes were measured for their surface expression of endothelial integrin subunits by flow cytometry. Median fluorescence intensity (MFI) was measured after forward versus side scatter data were tightly gated around, and normalised to, an isotype control. The bar chart shows the relative change in MFI of β3-HET compared to β3-WT ECs, or of β3-HET.NRP1Δcyto ECs compared to β3-WT.NRP1Δcyto ECs (means+s.e.m. from three independent experiments). Relative changes were deemed significant with a twofold change. Representative flow-cytometric histogram profiles are shown below for significantly changed integrins. (D) 70×10^5^ cells of the indicated genotypes were plated for 6 h on 10 μg/ml FN in six-well plates. Phase-contrast photographs were taken and cell surface areas were measured using ImageJ™ software. The bar chart represents mean (+s.e.m.) surface area quantified from multiple images (*n*≥50 cells per genotype). (E) ECs were firmly attached to FN-coated dishes and then imaged live for 15 h in low-serum medium ±VEGF. Individual cells were tracked every 10 min over this period using ImageJ™. The bar chart shows the EC migration speed of each of the indicated genotypes (mean+s.e.m. from three independent experiments; *n*=50 cells per condition). (F) ECs were plated onto FN-coated dishes overnight. After 3 h of starvation, a scratch wound was created and cells were incubated in low-serum medium ±VEGF for 24 h. The bar chart shows the percentage closure of the scratch ‘wound’ as a result of directed cell migration (means+s.e.m. from three independent experiments; *n*=27 for each condition). Representative images of scratch-wound closure at 24 h are shown below. Scale bar: 200 μm. Asterisks indicate statistical significance: *P<0.05; **P<0.01; ***P<0.001; nsd, not significantly different. Unpaired two-tailed *t*-test.

Prior to its description as a VEGF co-receptor, NRP1 was identified as a surface protein mediating cell adhesion (Takagi et al., 1995). Moreover, evidence has mounted to support a VEGFR2-independent role for NRP1 in regulating EC functions through an FA-dependent mechanism (Fantin et al., 2014; Raimondi et al., 2014; Seerapu et al., 2013). Because NRP1 is known to interact with a number of integrins (Fukasawa et al., 2007; Robinson et al., 2009; Valdembri et al., 2009), we first compared static cell adhesion between β3-WT, β3-HET, β3-WT;NRP1Δcyto and β3-HET;NRP1Δcyto ECs on saturating concentrations of various matrices. The only clear difference noted on saturating matrix concentrations in these assays was an expected reduced adhesion of β3-HET and β3-HET.NRP1Δcyto ECs to matrices containing VN, β3-integrin's canonical ligand (Fig. 3A). A more vigorous examination of adhesions (see Materials and Methods) over a range of matrix concentrations, however, uncovered subtle changes between the genotypes (Fig. 3B). Not surprisingly, compared to β3-WT and β3-WT;NRP1Δcyto ECs, β3-HET and β3-HET;NRP1Δcyto ECs showed reduced adhesion to VN over a range of concentrations tested. β3-WT;NRP1Δcyto EC adhesion to FN was, as expected (Valdembri et al., 2009), somewhat reduced compared to β3-WT ECs. Compared to their WT counterparts, β3-HET and β3-HET;NRP1Δcyto ECs exhibited reduced strength of adhesion to FN over the gradient of concentrations tested.

We therefore used flow-cytometry to examine the surface expression of various EC integrin subunits (Fig. 3C). Consistent with the adhesion data, αv- and β3-integrin levels were significantly higher in β3-WT and β3-WT;NRP1Δcyto ECs compared to their β3-HET counterparts. Whereas α1, β1 and β5 surface levels were unchanged, we noted a small increase in α2 surface expression in β3-HET ECs. Most notably, however, α5-integrin surface levels were lower in ECs expressing reduced levels of β3-integrin (Fig. 3C), which might partially account for their reduced adhesion to FN.

Our observations of VEGFR2-independent changes in ERK1/2 phosphorylation (a known regulator of integrin-mediated migration) that were nonetheless sensitive to alterations in NRP1 function in β3-HET but not β3-WT ECs prompted us to examine whether cell migration was differentially dependent on NRP1 upon reduced levels of β3-integrin expression. Because NRP1, α5β1-integrin and αvβ3-integrin all promote adhesion to FN (Hynes, 2002; Valdembri et al., 2009), we next set out to measure migration on this matrix component. In light of noted subtle differences in short-term adhesion to FN (and in surface expression of α5-integrin), we first tested long-term cell spreading of our four cell lines to 10 µg/ml FN, a saturating concentration which effectively promotes cell adhesion and migration (Clark et al., 1986; Sottile et al., 1998; Tvorogov et al., 2005). Cell spreading after 6 h was similar in all four genotypes (Fig. 3D). This, along with our static adhesion assays, which showed no differences between the genotypes on this concentration of the matrix, provided justification for examining random (Fig. 3E) and directed (Fig. 3F) migration in cells plated long-term (overnight) on this concentration of FN. Both types of migration assays revealed enhanced baseline and VEGF-induced migration in β3-HET ECs, compared to β3-WT ECs; unlike β3-WT ECs, β3-HET ECs were sensitive to NRP1 disruption.

### NRP1's localisation to focal adhesions is altered in β3-integrin-heterozygous endothelial cells

**Fig. 4.**
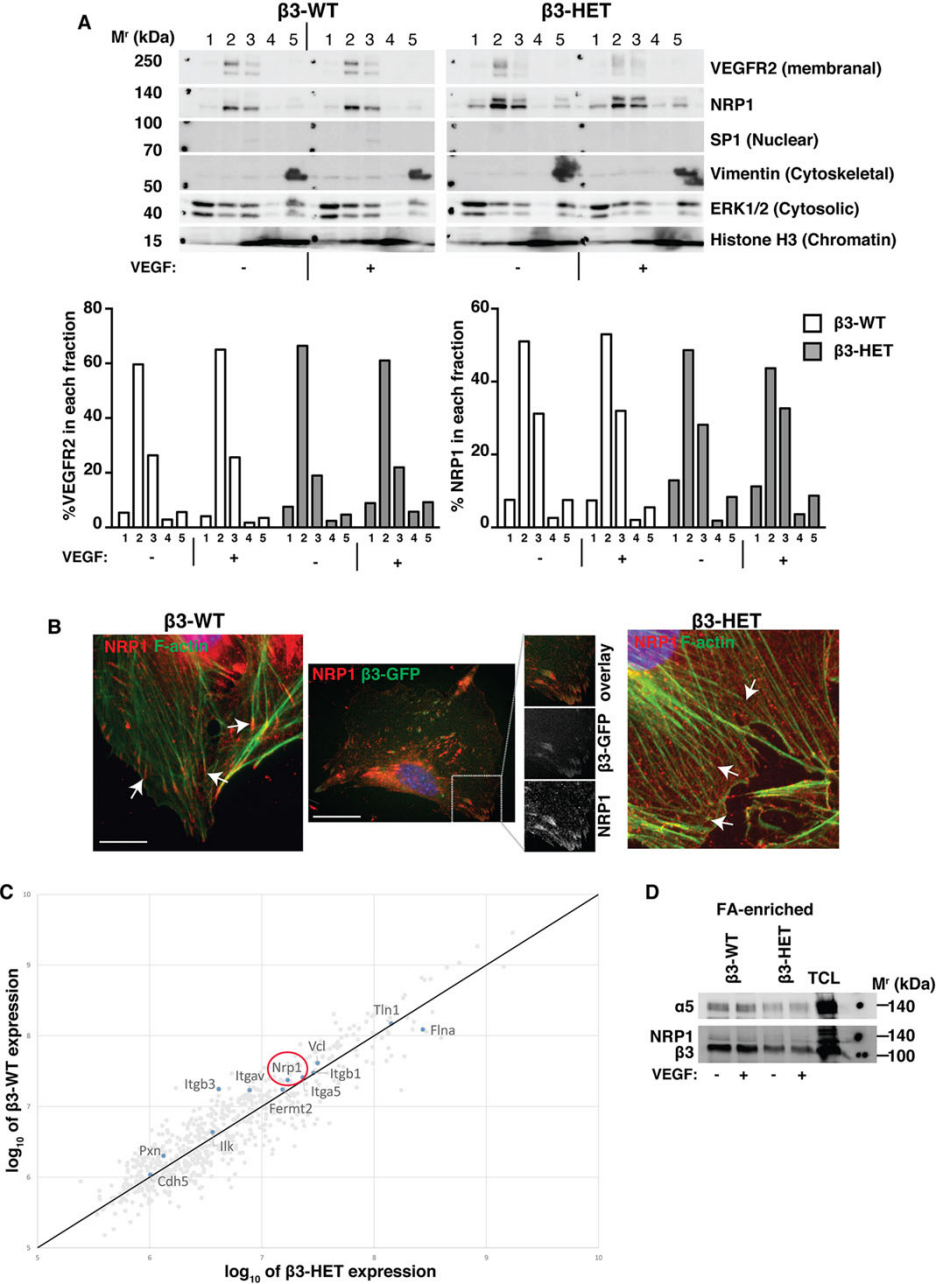
**NRP1's localisation in focal adhesions is altered in β3-integrin-heterozygous endothelial cells.** (A) β3-WT and β3-HET ECs were subjected to a cell-fractionation experiment following ±VEGF treatment for 10 min. Fractionated samples were then analysed by western blot for the indicated proteins. 1=cytoplasmic extract; 2=membrane extract; 3=soluble nuclear extract; 4=chromatin-bound nuclear extract; 5=cytoskeletal extract. Protein markers for each subcellular compartment were included as controls. Data are representative of two independent experiments. The bar charts represent the relative proportion (%) of VEGFR2 or NRP1 present in each fraction determined by ImageJ™ densitometry. (B) Left and right panels: β3-WT and β3-HET ECs were seeded overnight onto FN-coated glass coverslips and then stimulated with VEGF for 10 min. Cells were fixed and stained with phalloidin for filamentous actin (F-actin; green), and immunostained for neuropilin-1 (NRP1; red). Arrows point to the ends of actin filaments. Middle panel: β3-WT ECs were transfected with a β3-integrin-GFP construct (green) and seeded on FN-coated coverslips. 48 h later, cells were fixed and immunostained for NRP1 (red). Split-channel close-ups are shown to depict β3-integrin/NRP1 colocalisation. Scale bars: 10 μm (middle), or 20 μm. (C) ECs were allowed to adhere to FN-coated dishes for 90 min to establish ‘mature’ integrin-dependent focal adhesions (FAs). FAs were chemically cross-linked to the plates and cells were lysed with RIPA buffer. Non-cross-linked proteins and other cellular components were rinsed away under high-sheer flow. FA-enriched complexes were eluted, subjected to SDS-PAGE and then analysed by label-free, quantitative mass spectometry. The graphs represent log ratio plots of the proteomic data comparing unstimulated β3-WT ECs (*y*-axis) and β3-HET ECs (*x*-axis). Proteins above the diagonal line are higher in β3-WT ECs, whereas those below the line are higher in β3-HET ECs (*n*=3 samples per genotype). NRP1 is present in both adhesomes (red circle). (D) FA-enriched EC samples were processed as in C and then analysed by western blot for the indicated proteins. A total cell lysate (TCL) is shown for comparison. Data are representative of three independent experiments.

The findings described above suggested that aberrant integrin-directed migration, essential for pathological angiogenesis, correlates with the phenotypic difference in sensitivity to NRP1 perturbations that we observed in β3-HET mice. NRP1 is known to colocalise with a number of FA-associated proteins (Seerapu et al., 2013) and is involved in FA turnover. We therefore took a closer look at the structural and functional characteristics of mature endothelial FAs, focal points of cellular migration. We first examined NRP1's distribution in subcellular compartments after cell fractionation and immunoblotting. Other than the previously noted trend toward overexpression of the protein in β3-HET ECs, we found no obvious differences (Fig. 4A). We next immunolocalised NRP1 in cells plated on FN overnight. Because of the VEGF-dependence of our phenotypic angiogenic responses, we initially concentrated on VEGF-stimulated cells. In β3-WT ECs, NRP1 localised to mature FAs, which were found at the ends of filamentous actin (F-actin) fibres (Fig. 4B, left); as expected from previous studies (Robinson et al., 2009), NRP1 also colocalised with GFP-tagged β3-integrin in cDNA-expression-construct-transfected β3-WT ECs (Fig. 4B, middle). However, in VEGF-stimulated β3-HET ECs, NRP1 no longer localised to these sites (Fig. 4B, right).

To explore more precisely whether β3-integrin was required for the initial recruitment of NRP1 to FAs, we plated cells on FN for 90 min, which allows mature, β3-integrin-rich, adhesions to form (Schiller et al., 2011, 2013), and performed quantitative mass spectrometry of the FA-enriched endothelial adhesome from β3-WT and β3-HET ECs (Schiller et al., 2011) (Fig. 4C). For the present study, we focused our attention on known adhesome proteins. β3- and αv-integrins were, as expected, enriched in β3-WT adhesomes. Although many differences were noted between the two genotypes, the stoichiometry of many classical adhesome-related proteins (such as β1-integrin, vinculin, talin and integrin-linked kinase) was unchanged in β3-HET ECs. Most importantly, NRP1, as previously reported by others (Kuo et al., 2011; Schiller et al., 2011), was present within the adhesome of both genotypes. Immunoblotting of FA-enriched cellular lysates confirmed this finding (Fig. 4D); although the western blot (WB) signal for NRP1 was relatively weak, it was present in both β3-WT and β3-HET ECs. Together, these results suggest that β3-integrin is not essential for the initial localisation of NRP1 to mature FAs, but, rather, regulates its retention within FAs upon VEGF-stimulation.

### β3-integrin directs NRP1's control over FA remodelling

Given the noted changes in NRP1-dependent migration and retention within FAs following an angiogenic stimulus, we next investigated whether NRP1's association with FA proteins in general was altered in β3-HET ECs. We performed NRP1 immunoprecipitations on lysates from β3-WT and β3-HET ECs followed by label-free quantitative mass spectrometry, which highlighted a number of previously demonstrated NRP1 co-associations such as myosin-9, myosin-10 and filamin-A (Seerapu et al., 2013). Many VEGF-induced associations between NRP1 and FA-associated proteins were similar between the two genotypes, but β3-HET cells showed a number of significant changes in VEGF-induced interactions between NRP1 and cytoskeletal proteins involved with cell migration (Table 1), such as decreased interactions with filamin-A. These observations, coupled with changes in NRP1's mobilisation away from mature FAs upon VEGF-stimulation, suggested that NRP1-dependent changes in FA remodelling might be at the heart of phenotypic differences between β3-WT and β3-HET cells.

**Table 1.**
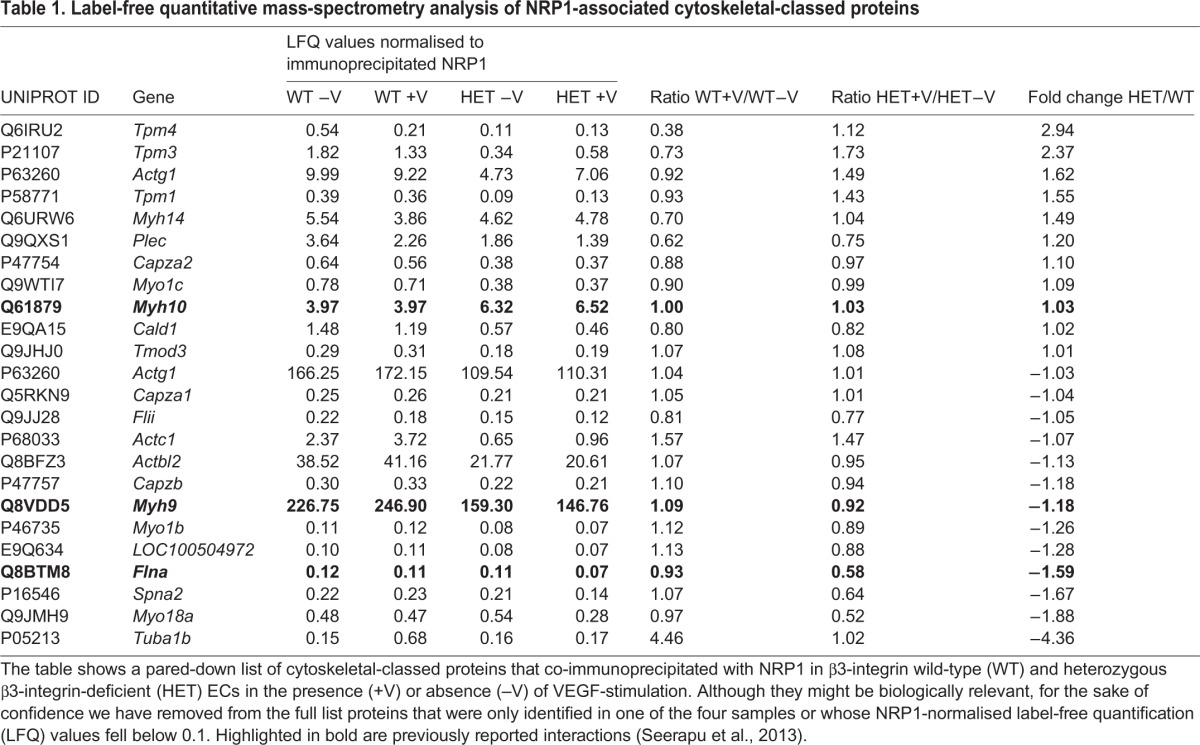
**Label-free quantitative mass-spectrometry analysis of NRP1-associated cytoskeletal-classed proteins**

**Fig. 5.**
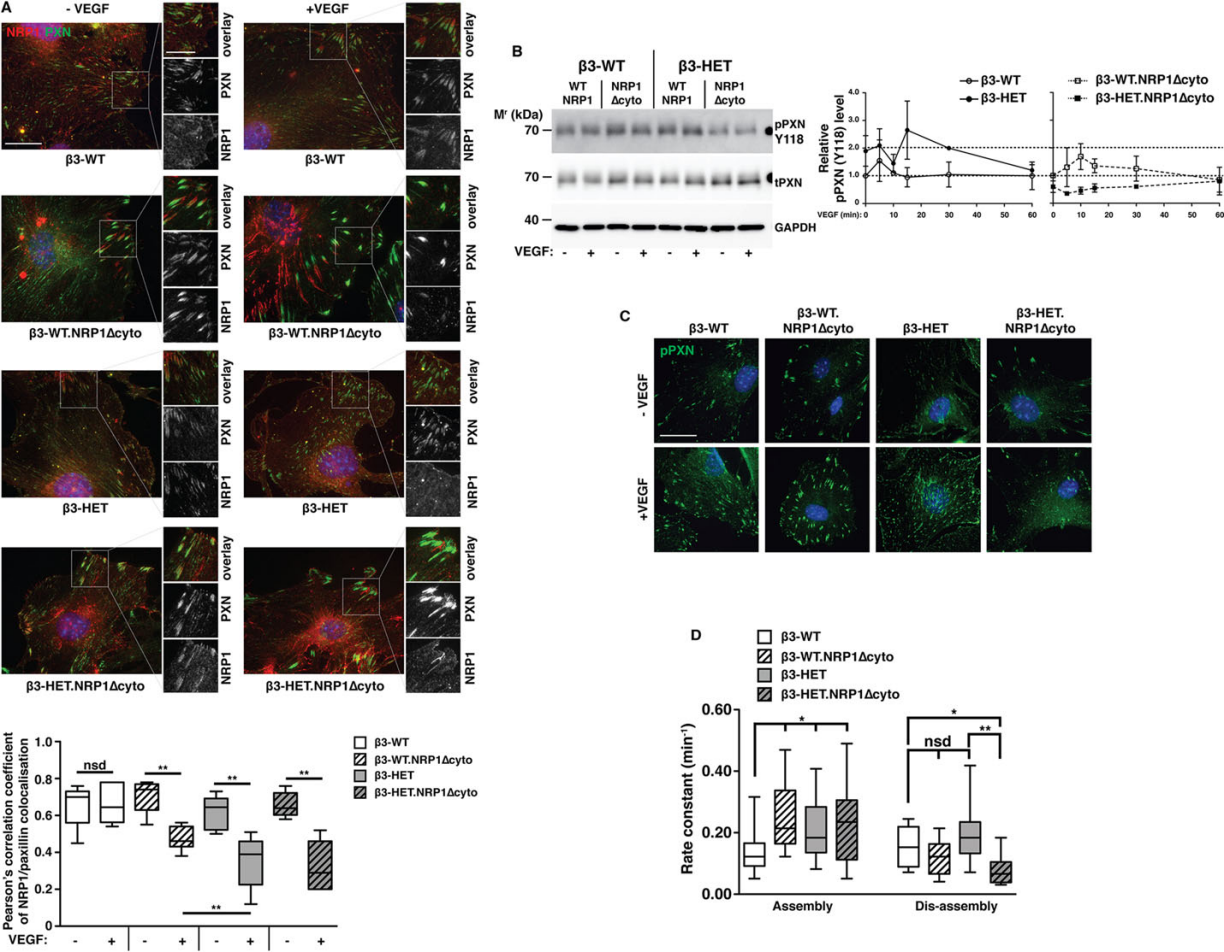
**Paxillin activation and focal adhesion disassembly are sensitive to NRP1 disruption in β3-integrin-heterozygous endothelial cells.** (A) ECs of the indicated genotypes were seeded overnight on FN-coated glass coverslips. After 3 h of starvation, cells were treated ±VEGF for 10 min in serum-free medium, then fixed and immunostained for total paxillin (PXN; green) and neuropilin-1 (NRP1; red). Split-channel close-ups are shown to depict PXN/NRP1 colocalisation. Scale bar: 20 μm. The box and whisker plot shows Pearson's correlation coefficient of PXN/NRP1 colocalisation in each of the indicated genotypes in the indicated regions as determined using the ImageJ™ coloc2 plugin (means±interquartile ranges and extreme values; *n*≥5 cells per genotype, over ≥three independent experiments). (B) Left panel: ECs of the indicated genotype were seeded overnight on FN. They were then starved for 3 h and treated ±VEGF for 10 min in serum-free medium. Cells were lysed and western blotted (WB) for levels of phosphorylated (p) and total (t) PXN. GAPDH served as a loading control. Data are representative of three independent experiments. Right panel: the graph shows densitometry of pPXN relative to tPXN, as determined by WB, over an extended VEGF time course (means±s.e.m. from ≥three independent experiments). (C) ECs were seeded overnight onto FN-coated glass coverslips and stained for pPXN. ECs were starved for 3 h and treated ±VEGF for 10 min in serum-free medium. Cells were fixed and immunostained for pPXN (green). Scale bar: 10 μm. (D) ECs were transfected with a PXN-GFP construct and seeded at a low density on FN-coated coverslips. 72 h later, cells were starved and then treated with VEGF in reduced-serum medium. Representative cells were then imaged live (every 2 min) on an inverted fluorescence microscope for 1 h to monitor focal adhesion (FA) remodelling. The front and back ends of individual FAs were tracked over this period to measure FA assembly and disassembly, using the ImageJ™ MTrack2 plugin. The box and whisker plot shows the rate of FA assembly or disassembly for each of the indicated genotypes (means±interquartile ranges and extreme values; *n*≥20 FAs per genotype, from ≥two independent experiments). Asterisks indicate statistical significance: *P<0.05; **P<0.01; nsd, not significantly different. Unpaired two-tailed *t*-test.

Therefore, as a marker of FAs, we turned our attention to examining interactions between NRP1 and PXN. As well as demarcating FAs, PXN plays an important role in EC motility and can be regulated by NRP1 (Raimondi et al., 2014). Immunocytochemistry of cells plated overnight on FN showed a predicted colocalisation of total PXN and NRP1 in non-VEGF-stimulated β3-WT ECs. This colocalisation was maintained after 10 min of VEGF-stimulation (Fig. 5A). NRP1/PXN colocalisation was also apparent in non-VEGF-stimulated β3-HET ECs, but it was lost upon VEGF-stimulation. A similar pattern was observed in β3-HET;NRP1Δcyto ECs, with β3-WT;NRP1Δcyto ECs showing only a small loss of NRP1/PXN colocalisation after VEGF-stimulation.

To pursue a potential β3-integrin-regulated NRP1 link with PXN activation, we also analysed the phosphorylation of PXN through tyrosine 118 by immunoblotting (Fig. 5B) and immunocytochemistry (Fig. 5C), and found a substantial reduction in PXN phosphorylation in β3-HET;NRP1Δcyto ECs, but not β3-WT;NRP1Δcyto ECs, suggesting that PXN activation is only NRP1-dependent when β3-integrin levels are suppressed. Moreover, it seems that FA disassembly only becomes substantially NRP1-dependent with reduced levels of β3-integrin: through the live tracking of GFP-PXN in transfected cells (Fig. 5D), we discovered that FA-assembly was faster in β3-HET, β3-WT;NRP1Δcyto and β3-HET;NRP1Δcyto ECs compared to β3-WT cells. Additionally, there was a trend toward increased FA-disassembly rates in β3-HET ECs. However, although FA-disassembly rates in β3-WT;NRP1Δcyto ECs were slightly reduced compared to their β3-WT counterparts, FA-disassembly rates were markedly retarded in β3-HET;NRP1Δcyto ECs, illustrating the increased role for NRP1 in these cells.

Overall, our data suggest that, in the presence of β3-integrin, EC migration is NRP1-independent; β3-integrin maintains NRP1 in FAs, thus ensuring a controlled migratory response to VEGF-stimulation. Reduced levels of β3-integrin lead to changes in FA remodelling and cell migration that are NRP1-dependent.

### Simultaneous depletion of both endothelial β3-integrin and NRP1 effectively inhibits already-established tumour growth and angiogenesis

**Fig. 6.**
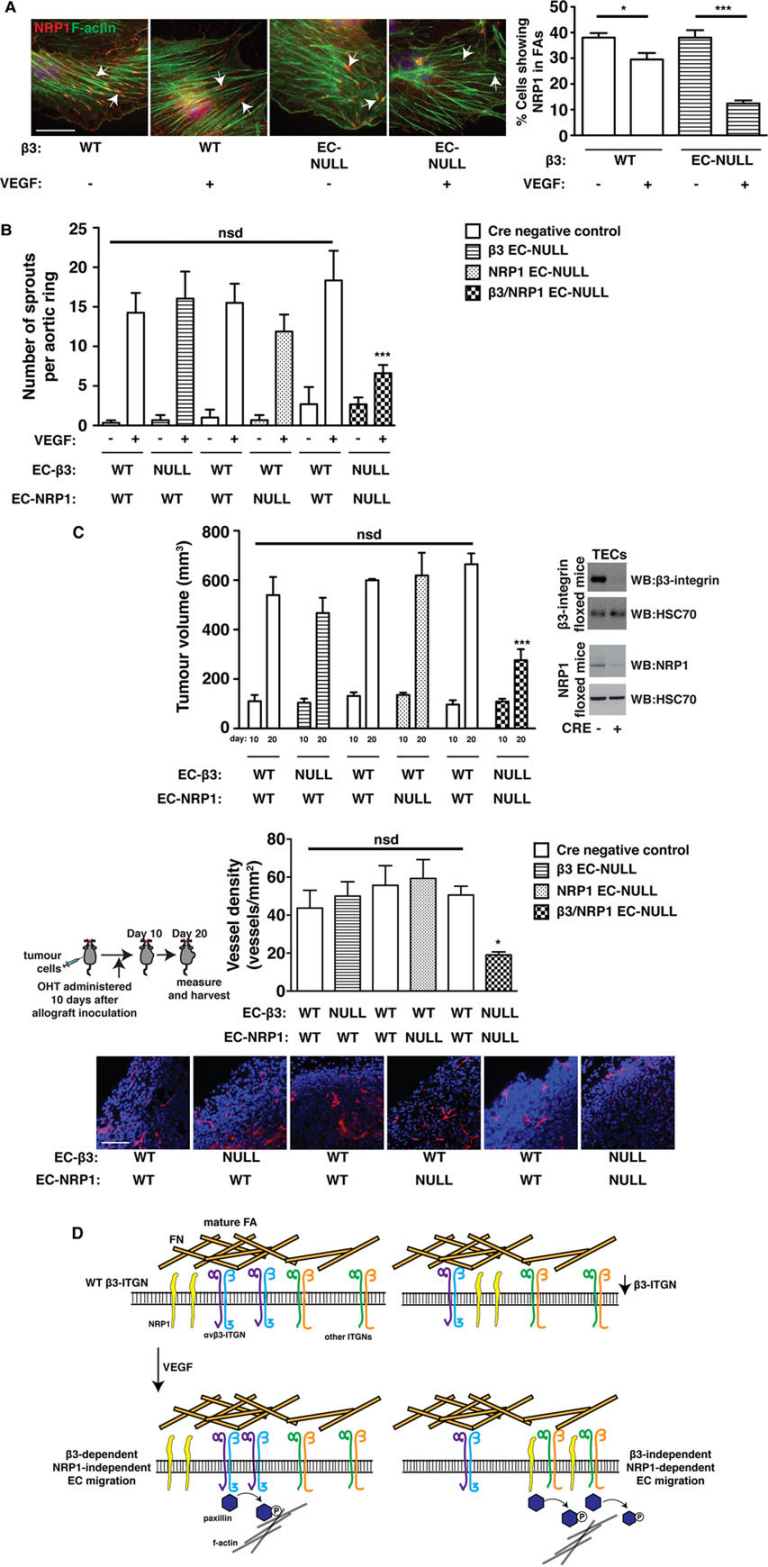
**Simultaneously depleting both β3-integrin and NRP1 blocks growth and angiogenesis in already-established tumours.** (A) Primary lung microvascular ECs were isolated from β3-floxed-Pdgfb-iCreER^T2^-negative and -positive animals. Tamoxifen (OHT) was administered after pure EC populations were achieved. Cells were then plated overnight on FN-coated glass coverslips. Cells were starved for 3 h and then treated ±VEGF for 10 min in serum-free medium. Cells were fixed and stained with phalloidin for F-actin (green), and immunostained for NRP1 (red). White arrows point to the ends of actin filaments. Scale bar: 20 μm. The bar chart shows the percentage of cells within the population showing NRP1 staining at the end of actin filaments (mean+s.e.m.; *n*≥50 cells per condition). (B) Microvessel sprouting of aortic ring explants of the indicated genotypes. Protein knockout in ECs was induced in culture with 1 μM OHT 4 days after VEGF-induced sprouting had been established. The bar chart shows the total number of microvessel sprouts per aortic ring after an additional 4 days of VEGF-stimulation (mean+s.e.m. from three independent experiments; *n*≥40 rings per genotype). (C) Tumour growth and angiogenesis were measured in animals of the indicated genotypes. Mice were injected subcutaneously with CMT19T cells and 10 days later OHT was administered. After an additional 10 days (20 days in total) tumours were harvested**.** Upper panel: the bar chart shows mean tumour volumes measured at days 10 and 20 (+s.e.m. of two or more independent experiments; *n*≥10 animals per genotype). The western blot to the right shows representative depletion of β3-integrin and NRP1 in tumour endothelial cells (TECs) isolated from Cre-positive animals, compared to Cre-negative littermate controls. Bottom panel: blood-vessel density in 20-day tumours was assessed by counting the total number of endomucin-positive vessels around the periphery (within 150 μm of the edge of the tumour) of midline bisected tumour sections. The bar chart shows mean vessel number per mm^2^ (+s.e.m.). Representative micrographs of endomucin staining (red) are shown below. Scale bar: 50 μm. Asterisks indicate statistical significance: *P<0.05; ***P<0.001; nsd, not significantly different. Unpaired two-tailed *t*-test. (D) Schematic representation of the hypothesised participation of NRP1 in focal adhesion (FA) remodelling and migration in β3-WT (left) and β3-suppressed (right) ECs. ITGN, integrin.

The identification of the mechanisms underlying increased sensitivity to NRP1 disruption in ECs with reduced β3-integrin expression should enable the rational design of intervention strategies to improve anti-angiogenic outcomes in patients with advanced cancers. To provide evidence in support of this idea, we performed proof-of-concept studies in β3-integrin/NRP1-double-floxed mice crossed to OHT-inducible-Pdgfb-iCreER^T2^ transgenics. First, though, we confirmed the same mechanistic principle described above in primary lung ECs acutely depleted of β3-integrin (Fig. 6A). We observed NRP1 expression at the ends of F-actin in OHT-treated β3-WT cells with and without VEGF-stimulation, but not in the majority of OHT-treated β3-KO ECs after VEGF treatment. We then initiated VEGF-induced microvessel sprouting in aortic rings isolated from: (1) β3-floxed mice with and without Pdgfb-iCreER^T2^; (2) NRP1-floxed mice with and without Pdgfb-iCreER^T2^; or (3) double-floxed mice with and without Pdgfb-iCreER^T2^. OHT was administered to all rings after 4 days of sprouting, and microvessels were enumerated 4 days later. Only in rings from double-floxed Pdgfb-iCreER^T2^-positive animals was further sprouting significantly inhibited (Fig. 6B). Finally, we performed intervention CMT19T allograft studies by establishing vascularised tumours in these same animals. After 10 days of growth, all animals were administered OHT and tumours were allowed to grow for another 10 days. In concordance with the intervention aortic ring studies, further tumour growth and angiogenesis were significantly inhibited in double-floxed Pdgfb-iCreER^T2^-positive animals, but not in any of the other genotypes (Fig. 6C).

## DISCUSSION

We previously showed that the total loss of β3-integrin expression sensitises angiogenesis to NRP1 inhibition by siRNA or small peptides (Robinson et al., 2009). This finding suggested to us, even at the time, that an anti-angiogenic approach that simultaneously targeted both molecules might offer therapeutic benefit to patients with advanced cancers where traditional mono-target strategies were largely failing; a concept strengthened further by our recent findings demonstrating only transient inhibition of tumour growth and angiogenesis after long-term depletion of endothelial β3-integrin expression (Steri et al., 2014). Testing this notion, however, requires that we have a deeper mechanistic understanding of, at the very least, how the dual targeting affects its outcome. Achieving this understanding necessitated moving away from β3-KO animals, which are often criticised for sustaining angiogenesis through developmental upregulation of VEGFR2, to a more subtle, albeit global, alteration in β3-integrin expression – β3 heterozygosity.

Like their knockout counterparts (Robinson et al., 2009), β3-HET mice display increased sensitivity to perturbations in NRP1 expression and we extend this finding to include NRP1 function, as assayed through the deletion of its cytoplasmic tail. In marked contrast to β3-KO animals, however, this phenomenon does not occur through increased interactions in ECs between VEGFR2 and NRP1 (Fig. 2). Rather, the work we present here guides us toward a newly identified mechanism for β3-integrin function whereby it regulates the retention of NRP1 within mature FAs upon VEGF-stimulation (Fig. 5).

A VEGFR2-independent role for NRP1 in regulating EC migration is gaining ground. NRP1's cytoplasmic domain is known to promote FN fibrillogenesis in arterial ECs by regulating the trafficking of activated α5β1-integrin (Valdembri et al., 2009), and NRP1 is involved in ABL1-mediated PXN activity in human dermal ECs (Raimondi et al., 2014) on FN. Crucially, however, here we report that NRP1's involvement in EC migration, at least in microvascular ECs, is regulated by β3-integrin. Endothelial NRP1 seems to play no role in pathological angiogenesis in β3-WT mice (Figs 1 and 6). Although this idea has been alluded to in a number of studies (Fantin et al., 2011, 2013; Lanahan et al., 2013; Robinson et al., 2009), we fully extend this conclusion to primary tumour growth and angiogenesis. In the models that we have employed, we demonstrate, categorically, that NRP1 perturbations alone do not disrupt pathological angiogenesis. When coupled to reductions in β3-integrin levels, however, NRP1's participation in angiogenesis becomes essential. Equally, reductions in β3-integrin expression without concomitant changes in NRP1 do not drastically alter tumour angiogenesis (particularly in already-established tumours). This suggests that there is redundancy built into the system such that the presence of either molecule in mature FAs is sufficient to mediate adequate PXN activity.

We hypothesise a default system in wild-type microvascular ECs in which αvβ3-integrin's molecular associations at mature FAs predominate and prevent NRP1 from fully participating in FA dynamics. αvβ3-integrins are known to immobilise in static FAs (Zamir et al., 2000) and it seems that they help to sequester NRP1 at these sites, even after VEGF-stimulation, which prevents it from activating PXN. In contrast, the long-term suppression of β3-integrin triggers an escape pathway whereby PXN activation and EC migration become dependent on NRP1 function; mobilisation of NRP1 away from mature/static FAs allows it to direct PXN activity (Fig. 6D). The idea of differential FA dynamics dependent on which integrin subclasses are engaged with an FN-rich ECM is not a new concept (see Truong and Danen, 2009, for example). However, this is the first time that differential NRP1 localisation and function have been linked to these changes.

The role that NRP1 plays in mediating VEGF-induced ERK phosphorylation seems to be controversial and dependent on the EC source and method employed to elicit NRP1 inhibition. Although Pan et al. (2007) found that anti-NRP1 monoclonal antibodies (mAbs) had no effect on VEGF-dependent ERK phosphorylation in human umbilical vein endothelial cells (HUVECs), Murga et al. (2005) demonstrated reduced VEGF-induced ERK phosphorylation in these cells after *NRP1* siRNA knockdown. Lanahan et al. (2013) found that the loss of NRP1's cytoplasmic tail reduced VEGF-dependent ERK phosphorylation in both heart and arterial ECs. Raimondi et al. (2014) report that *NRP1* siRNA knockdown impairs ERK phosphorylation in human dermal microvascular endothelial cells (HDMECs). We report here that the deletion of NRP1's cytoplasmic tail does not affect VEGF-induced ERK phosphorylation in WT microvascular ECs (Fig. 2). These apparent discrepancies need addressing and are the focus of ongoing research in our laboratory.

It is particularly important to address these discrepancies given our finding that β3-integrin's regulation of NRP1 function is dependent on the presence of VEGF, even when NRP1's regulation of VEGFR2 is unchanged. This is important clinically because it provides a therapeutic opportunity to enhance the efficacy of current strategies that largely focus on manipulating the VEGF-VEGFR2 pathway, which is linked with significant side-effects and prone to treatment resistance (Ebos and Kerbel, 2011). We now have the chance to affect VEGF-dependent angiogenesis in an apparently VEGFR2-independent manner.

This study provides proof-of-concept that a dual-combative αvβ3-integrin/NRP1 targeting approach offers a clinically beneficial way of treating advanced solid cancers. Small-molecule inhibitors directed against NRP1 are currently under development and we hope that these can soon be tested alongside existing or new αvβ3-integrin antagonists, with the caveat that both molecules are expressed by multiple cell types that contribute to tumour growth and angiogenesis, including platelets, and off-target (i.e. non-EC) effects will have to be examined carefully; although we can rule out their contribution to the EC-double-KO intervention studies (Fig. 6), these other cells types might be contributing to β3-HET angiogenic responses. Nonetheless, we provide a strong mechanistic foundation for understanding the molecular basis of how a dual-targeted approach against these two endothelial molecules might meet with success. This will allow us, in the meantime, to more fully explore the long-term durability of such an approach when applied to additional clinically relevant scenarios. Moreover, detailed further analysis and extension of our mass spectrometric studies in ECs will allow us to fully explore how differential adhesion dynamics, mediated by distinct integrin-ECM interactions, result in the formation of unique signalling platforms that can be exploited to manipulate angiogenic responses.

## MATERIALS AND METHODS

### Reagents

VEGF-A^164^ was made in-house according to the method published by Krilleke et al. (2007). All chemicals were from Sigma-Aldrich (Poole, UK) unless otherwise indicated.

### Animals

All animals were on a mixed C57BL6/129 background. Littermate controls were used for all *in vivo* experiments. All animal experiments were performed in accordance with UK Home Office regulations and the European Legal Framework for the Protection of Animals used for Scientific Purposes (European Directive 86/609/EEC).

### *In vivo* tumour growth assays

Mouse melanoma (B16F0, ATCC; mycoplasma free) or mouse lung carcinoma (CMT19T, CR-UK Cell Production; mycoplasma free) cells (1×10^6^) were injected subcutaneously in the flank of experimental and littermate-control mice. 12-20 days after injection, mice were killed, tumour sizes measured and tumour samples were fixed in 4% paraformaldehyde for histological analysis. For prevention studies in Pdgfb-iCreER^T2^ mice (Fig. 1, supplementary material Fig. S1), slow release (5 mg, 21-day release) tamoxifen pellets (Innovative Research of America, Sarasota, FL) were implanted subcutaneously into the scruff of the neck 3 days prior to tumour cell injection. For intervention studies (Fig. 6), pellets were implanted after 10 days of initial tumour growth. Tumour volumes were calculated according to the formula: length×width^2^×0.52.

### Immunohistochemical analysis of tumour sections

At 24-h post-fixation, tumours were bisected at the midline and embedded in paraffin (cut face toward blade) and 5-μm sections were prepared. Immunostaining was then performed with sodium-citrate antigen retrieval as described previously (Reynolds et al., 2002). Images were acquired on an Axioplan (Zeiss, Cambridge, UK) epifluorescent microscope and tissue area was quantified using ImageJ™ software available at the National Institutes of Health website. Primary antibodies were: rat anti-endomucin (clone V.7C7, used at 1:500, Santa Cruz Biotechnology, Santa Cruz, CA); rabbit anti-CD146 (clone EPR3208, used at 1:500, Abcam, Cambridge, UK). Secondary antibodies were: donkey anti-rat Alexa-Fluor^®^-594 and donkey anti-rabbit Alexa-Fluor^®^-488 conjugates (Invitrogen, Paisley, UK), both used at 1:500.

Blood-vessel density was assessed by counting the total number of endomucin-positive vessels per mm^2^ across entire midline tumour sections from age-matched, size-matched tumours. For tumour sections from the intervention studies (Fig. 6), vessels around the perimeter of the sections were counted in order to avoid the necrotic centres of tumours.

### Mouse tumour endothelial cell isolation

Tumour ECs were isolated and analysed by western blot as previously described by Steri et al. (2014).

### *Ex vivo* aortic ring assay

Thoracic aortae were isolated from 6- to 9-week-old adult mice and prepared for culture as described extensively by Baker et al. (2012). Protein knockout in ECs was induced in culture with 1 μM 4-hydroxytamoxifen (OHT). Where indicated, VEGF was added at 30 ng/ml. Microvessel growth of aortic rings was quantified after 6-10 days. For the intervention study, protein knockout in ECs was induced in culture with 1 μM OHT 4 days after VEGF-induced sprouting had been established, and the microvessel sprouts were quantified after an additional 4 days of VEGF-stimulation.

### Mouse lung microvascular endothelial cell isolation and culture

Primary mouse lung ECs were isolated from adult mice as described previously by Reynolds and Hodivala-Dilke (2006). To induce target gene deletion in Pdgfb-iCreER^T2^-floxed cell lines, cells were grown for 48 h in medium supplemented with 500 nM OHT.

For immortalisation, cells were treated with polyoma-middle-T-antigen (PyMT) retroviral transfection as described previously by Robinson et al. (2009). Briefly, PyMT conditioned medium was collected, filter sterilised using a 0.45 μm filter, and stored at −80°C until use. Following two rounds of CD102-positive selection, primary ECs in six-well plates were treated with the preserved PyMT conditioned medium supplemented with 8 μg/ml polybrene for 6 h at 37°C. PyMT conditioned medium was removed and replaced with complete growth medium. This same procedure was repeated the following day. Cells were observed and passaged for 4 weeks to ensure their immortalisation. Subsequently, they were maintained in a 1:1 mixture of DMEM low glucose:Ham's F12 nutrient mixture (Invitrogen) supplemented with 0.1 mg/ml heparin and 10% FBS, and used between passages 5-20. Cells were routinely checked by flow cytometry for surface expression of ICAM2, CD31 and VECAD (see supplementary material Fig. S3) to ensure that they retained their normal EC characteristics. Cells were also routinely checked for their ability to survive extended periods of confluency, which indicates absence of transformation (May et al., 2005).

For experimental analyses, tissue culture plates and flasks were coated overnight at 4°C with one or more of the following, as specified below: 0.1% gelatin (type A from porcine skin, ∼300 g bloom), Purecol (COLI) (Nutacon B.V., The Netherlands), human plasma fibronectin (FN) (Millipore, Watford, UK) and mouse multimeric vitronectin (VN) (Patriecell Ltd, Nottingham, UK).

### Western blot analysis

For the analysis of VEGFR2, NRP1, β3-integrin, ERK1/2, p130cas and FAK, ECs were seeded at 2×10^5^ cells per well in six-well plates coated with 0.1% gelatin, 10 μg/ml FN, 10 μg/ml COLI and 2 μg/ml VN. For paxillin analysis, ECs were seeded at the same density, but on plates coated with only 10 μg/ml FN in PBS. 24 h later, cells were starved for 3 h in serum-free medium (OptiMEM^®^; Invitrogen). VEGF was then added to a final concentration of 30 ng/ml and cells were lysed at the indicated times (see main text) in EB (3% SDS, 60 mM sucrose, 65 mM Tris-HCl pH 6.8). 15-30 μg of protein from each sample was loaded onto 8-10% polyacrylamide gels. For paxillin analysis, samples were loaded onto a 4-12% gradient gel for better resolution. The protein was transferred to a nitrocellulose membrane and incubated for 1 h in 5% milk powder/PBS plus 0.1% Tween-20 (PBSTw), followed by an overnight incubation in primary antibody diluted 1:1000 in 5% bovine serum albumin (BSA)/PBSTw at 4°C. The blots were then washed 3× with PBSTw and incubated with the relevant horseradish peroxidase (HRP)-conjugated secondary antibody (Dako) diluted 1:2000 in 5% milk/PBSTw, for 1 h at room temperature. Chemiluminescence was detected on a Fujifilm LAS-3000 darkroom (Fujifilm UK Ltd, Bedford, UK). Antibodies (all used at 1:1000 and purchased from Cell Signaling Technology, unless noted otherwise) were: anti-phospho (Y1175) VEGFR2 (clone 19A10); anti-VEGFR-2 (clone 55B11); anti-Neuropilin-1 (cat. no. 3725); anti-β3-integrin (cat. no. 4702); anti-phospho (Thr202/Tyr204) p44/42 MAPK Erk1/2 (clone D13.14.4E); anti-total p44/42 MAPK Erk1/2 (clone 137F5); anti-phospho (Y410) p130cas (cat. no. 4011); anti-p130cas (cat. no. 610271, BD Biosciences, Oxford); anti-phospho (Y407) FAK (#OPA1-03887, ThermoScientific); anti-FAK (cat. no. 3285); anti-HSC70 (clone B-6, Santa Cruz Biotechnology); anti-phospho (Y118) paxillin (cat. no. 2541); anti-paxillin (ab32084; Abcam); anti-GAPDH (14C10, cat. no. 2118); anti-SP1 (cat. no. 5931); anti-histone H3 (D1H2, cat. no. 4499); anti-vimentin (D21H3, cat. no. 5741).

### Flow cytometry

For flow-cytometric analysis, cells were trypsinised, resuspended in FACS buffer (1% FBS in PBS+1 mM CaCl_2_+1 mM MgCl_2_) and labelled with one of the following antibodies (all used at 1:200 and, unless stated otherwise, purchased from eBioscience, Hatfield, UK): PE-anti-mouse Flk1, PE-anti-mouse CD49a (Cambridge Bioscience, Cambridge, UK); PE-anti-mouse CD49b; PE-anti-mouse CD49e; PE-anti-mouse CD51; PE-anti-mouse CD29; PE-anti-mouse CD61; PE-anti-mouse integrin beta 5; PE-anti-mouse CD31; FITC-anti-mouse ICAM2; PE-anti-mouse VECAD; appropriate PE/FITC-labelled isotype-matched controls were from eBioscience. In the case of Flk1 analysis, cells were stimulated with 30 ng/ml VEGF at 37°C over a 60-min time course before trypsinisation.

### Immunoprecipitation assay

Cells were grown to 80-90% confluency in 15-cm dishes coated with 10 μg/ml FN in PBS. After starvation in OptiMEM^®^ for 3 h, cells were stimulated with 30 ng/ml VEGF for 10 min (+VEGF), or for the indicated times, at 37°C. Cells were then placed on ice, washed two times with PBS, and lysed in 0.5 ml/plate of RIPA buffer (20 mM Tris pH 7.4, 50 mM NaCl, 0.1% SDS, 1% Triton, 1% Deoxycholate, 1% NP40) containing PMSF (∼1 mM) and Halt^®^ Protease and Phosphatase inhibitor (1:100). Lysates were centrifuged at 12,000 ***g*** for 10 min at 4°C. 400 μg of total protein from each sample was immunoprecipitated by incubating them with protein-G Dynabeads^®^ (Invitrogen) coupled to a rabbit-anti-mouse-VEGFR2 antibody (clone 55B11, Cell Signaling Technology) for the VEGFR2 immunoprecipitation (IP), or a goat anti-mouse Neuropilin-1 antibody (AF566, R&D Systems) for the NRP1 IP, on a rotator overnight at 4°C. Immunoprecipitated complexes were washed three times with 0.2 ml of RIPA buffer, and once in PBS, before being added to, and boiled in, 1× NuPAGE^®^ sample reducing agent (Life Technologies), ready for western blotting or mass spectrometry analysis.

### Adhesion assays

#### Static adhesion

96-well plates were coated overnight at 4°C with 10 μg/ml COLI, 10 μg/ml FN, 10 μg/ml laminin-I (LN) or 2 μg/ml VN in PBS, or a mixture (MIX) containing 10 μg/ml COLI, 10 μg/ml FN and 2 μg/ml VN in 0.1% gelatin was also used. The wells were then washed with PBS, and blocked for 1 h at room temperature (RT) with 1% BSA in PBS, before a final wash in PBS. Prior to seeding, cells were starved for 3 h in Opti-MEM^®^, trypsinised and resuspended in serum-free OptiMEM^®^. They were then seeded in serum-free OptiMEM^®^ at a concentration of 1×10^4^ cells/well for 90 min at 37°C. Plates were washed three times gently by immersion in a bucket of PBS, and any excess volume was removed. Wells were stained with methylene blue for 30 min, washed for 15 min under running water and air-dried. Dye was extracted with 50% ethanol:50% 0.1 N HCl and the absorbance of each well was measured at 610 nm.

#### Adhesion on various matrix concentrations

96-well plates were coated overnight at 4°C with serial dilutions of VN or FN. The wells were then washed with PBS, and blocked for 1 h at RT with 1% BSA in PBS. Prior to seeding, cells were starved for 3 h in Opti-MEM^®^, trypsinised and resuspended in serum-free OptiMEM^®^. They were then seeded in serum-free Opti-MEM^®^ at a concentration of 3×10^4^ cells/well for 90 min at 37°C. Plates were tapped vigorously on the bench top and wells were washed thoroughly using a multi-channel pipette. Wells were stained with methylene blue for 30 min, washed for 15 min under running water and air-dried. Dye was extracted with 50% ethanol:50% 0.1 N HCl and the absorbance of each well was measured at 610 nm.

### Random-migration assay

ECs were starved in OptiMEM^®^ for 3 h, trypsinised and seeded at 1.5×10^4^ cells/well in 24-well plates coated with 10 μg/ml FN in PBS, and allowed to adhere for 3 h. The media was then replaced with OptiMEM^®^+2% FBS, and half of the wells were supplemented with 30 ng/ml VEGF. One phase-contrast image/well was taken live every 10 min in a fixed field of view using an inverted Axiovert (Zeiss) microscope for 15 h at 37°C and 4% CO_2_. Individual cells were then manually tracked using the ImageJ™ cell tracking plugin, and the speed of random migration was calculated in μm moved/hour.

### Wound-closure assay

ECs were seeded at 4×10^5^ cells/well in six-well plates coated with 10 μg/ml FN in PBS, and cultured until the next day, by which time they had reached confluency. Cells were serum starved for 3 h in OptiMEM^®^ before scratching the confluent monolayer with a P200 pipette tip. Phase-contrast images of scratches were then captured and the media was changed to OptiMEM^®^ containing 30 ng/ml VEGF. After 24 h, cells were fixed for 10 min with 4% formaldehyde and scratches were imaged again. The degree of scratch-wound closure was quantified by measuring the gap between cells in three areas per field using Axiovision (Zeiss) software, taking an average, and calculating the length of change between time points.

### Immunocytochemistry

Either primary or immortalised ECs were seeded at 1.5×10^5^ cells/well in six-well plates on acid-washed and oven-sterilised glass coverslips, coated with 10 μg/ml FN in PBS and cultured until the next day. Cells were starved for 3 h in serum-free OptiMEM^®^, and either stimulated with 30 ng/ml VEGF at 37°C for 10 min (+VEGF), or not at all (−VEGF). Cells were then fixed in 4% formaldehyde for 10 min, washed in PBS, permeabilised with 0.5% NP40 in PBS, blocked in 0.1% BSA+0.2% Triton in PBS, and incubated with primary antibody diluted 1:100 in PBS for 1 h at RT. After further PBS washes, cells were incubated with the relevant Alexa-Fluor^®^-conjugated secondary antibody (Invitrogen) diluted 1:500 in PBS for 45 min at RT. Coverslips were washed in PBS again before they were mounted on slides with Prolong^®^ Gold containing DAPI (Invitrogen). To stain for filamentous-(F) actin, Alexa-Fluor^®^-568–phalloidin (Invitrogen) was used 1:300 in PBS at the secondary-antibody incubation stage. To look at β3-integrin fluorescently, 1×10^6^ ECs were transfected with a GFP-tagged β3-integrin cDNA expression construct (provided by Dr Maddy Parsons, King's College London, London, UK) by nucleofection prior to seeding on coverslips at 1.5×10^5^ cells/well. Antibodies (all used at 1:100) were: anti-phospho (Y118) paxillin (Cell Signaling Technology, cat. no. 2541); anti-paxillin (ab32084, Abcam); anti-neuropilin-1 (AF566, R&D Systems). NRP1-PXN colocalisation was quantified using the Coloc2 ImageJ™ plugin to determine the Pearson's correlation coefficient.

### Cell fractionation assay

ECs were seeded in plates coated with 0.1% gelatin, 10 μg/ml FN, 10 μg/ml COLI and 2 μg/ml VN. 24 h later, cells were starved for 3 h in serum-free OptiMEM^®^, and either stimulated with 30 ng/ml VEGF or not. Cells were then trypsinised and centrifuged at 500 ***g***. Cell fractionation was carried out following the ‘Subcellular protein fractionation kit for cultured cells' (ThermoScientific) protocol exactly, and samples were prepared for western blotting.

### Focal-adhesion enrichment

ECs were starved in serum-free OptiMEM^®^ for 3 h and seeded at 6×10^6^ cells/plate in 10-cm plates that were previously coated with 10 μg/ml FN in PBS overnight at 4°C and blocked in 1% BSA in PBS for 1 h at RT. Cells were allowed to adhere for 90 min to allow for mature FAs to form and either stimulated with 30 ng/ml VEGF at 37°C for 10 min (+VEGF) or not at all (−VEGF). Cells were washed in PBS+1 mM CaCl_2_+1 mM MgCl_2_ (PBS^++^) and incubated with 0.5 mM Dithiobis(succinimidyl propionate) (DSP) and 0.05 mM 1,4-di-[30-(20-pyridyldithio)-propionamido] butane (DPP) diluted in PBS^++^ for 5 min to cross-link FAs to the plate. This reaction was quenched with 1 M Tris-HCl pH 7.5 before cells were lysed in RIPA for 30 min on ice with occasional agitation. RIPA was collected without scraping, and the plates were blasted with a high-sheer flow jet of distilled water to remove cell debris. Cross-linked proteins were eluted with 2 ml dithiothereitol (DTT) buffer (25 mM Tris-HCl pH 7.5, 10 mM NaCl, 0.1% SDS, 100 mM DTT) for 1 h at 60°C in a sealed and humidified chamber. 8 ml of acetone was added to this solution and left overnight at −20°C to allow the proteins to precipitate. Samples were then centrifuged at 13,000 ***g*** for 40 min, and the acetone layer removed. The pellet was resuspended in EB (see above) ready for western-blot or mass-spectrometry analysis.

### Focal-adhesion tracking

1×10^6^ ECs were transfected with a GFP-tagged paxillin cDNA expression construct (kindly provided by Dr Maddy Parsons, King's College London, London, UK) and a fraction of these were seeded on acid-washed and oven-sterilised glass coverslips, coated with 10 μg/ml FN in PBS, in wells of a six-well plate. Cells were cultured for ∼48 h before they were starved in serum-free OptiMEM^®^ for 3 h. In turn, individual coverslips were separately transferred to OptiMEM^®^+2% FBS and 30 ng/ml VEGF was added. An Axiovert (Zeiss) inverted microscope was then used to take live images of the GFP-paxillin-positive focal adhesions in a selected field of view every 2 min for 1 h at 37°C+4% CO_2_. Assembly and disassembly was quantified by manually tracking leading and trailing edges of FAs using the MTrackJ plugin for ImageJ™.

### Mass-spectrometry analysis

Mass spectrometry was carried out by the Fingerprints Proteomics Facility, Dundee University, Dundee, UK as per Schiller et al. (2011). Peptides were identified and quantified using MaxQuant software using the Andromeda peptide database. To achieve label-free quantitative results, three biological repeats were pooled and each of these pooled samples was analysed via three technical repeats through the spectrometer.

### Statistical analysis

Significant differences between means were evaluated by Student's *t*-test. *P*<0.05 was considered statistically significant. For flow cytometric analysis of integrins, relative differences were deemed significant if they were greater than twofold.

## Supplementary Material

Supplementary Material
