# Supplementary material for: Suppression of β3-integrin in mice triggers a neuropilin-1-dependent change in focal adhesion remodelling that can be targeted to block pathological angiogenesis
Source: Dis Model Mech. 2015 Sep 1;8(9):1105–19. doi: 10.1242/dmm.019927 (PMC4582102; doi:10.1242/dmm.019927)
Supplement: Supplementary Material [file supp_8_9_1105__index.html]

Supplementary Material 

# Suppressing β3-integrin triggers a neuropilin-1 dependent change in focal adhesion remodelling that can be targeted to block pathological angiogenesis

## DMM019927 Supplementary Material

- Supplementary Material
