# Supplementary material for: Suppression of β3-integrin in mice triggers a neuropilin-1-dependent change in focal adhesion remodelling that can be targeted to block pathological angiogenesis
Source: Dis Model Mech. 2015 Sep 1;8(9):1105–19. doi: 10.1242/dmm.019927 (PMC4582102; doi:10.1242/dmm.019927)
Supplement: Supplementary Material [file supp_019927_DMM019927supp.pdf]

Supplementary Figures

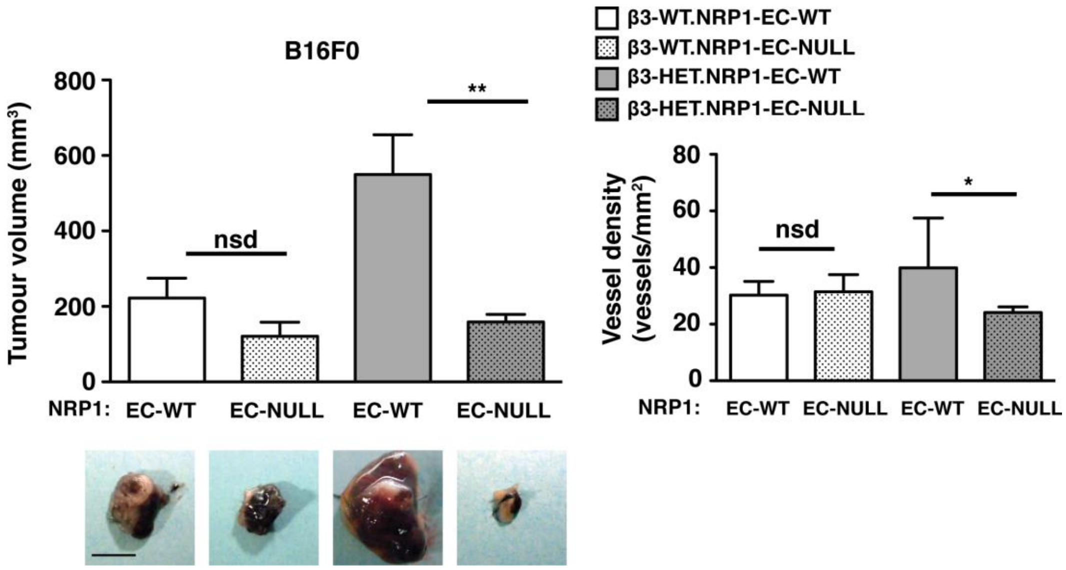

Supplementary Figure 1: B16F0 tumour growth and angiogenesis in β3-integrin-heterozygous mice are sensitive to NRP1 perturbations. Tumour growth and angiogenesis were measured in animals of the indicated genotypes. **Left panels**. Mice were given subcutaneous injections of B16F0 tumour cell lines. To generate NRP1-EC-KO (EC-Null), 21-day slow-release OHT pellets were administered 3-days prior to tumour cell injection. OHT-treated Cre-negative (EC-NRP-WT) littermates served as controls. Tumour volumes were measured after 12 days of growth (mean +SEM of 3 independent experiments; n≥10 animals per genotype). Representative pictures of tumour macroscopic appearances are shown. Scale bar = 10mm. **Right panels**. Blood vessel density was assessed by counting the total number of endomucin-positive vessels across tumour sections (mean +SEM; n≥10 sections per genotype).

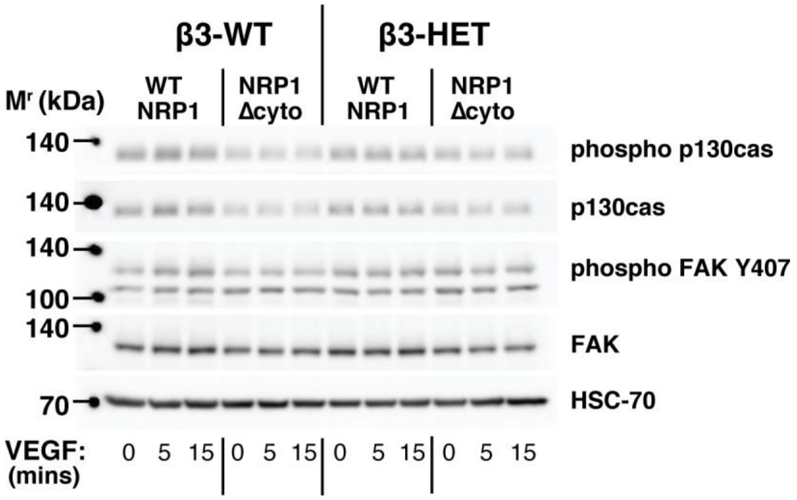

Supplementary Figure 2: No changes in the expression/activity of proteins involved in NRP1-regulated VEGF signalling. ECs were seeded on a complex matrix containing gelatin, collagen, fibronectin and vitronectin to preserve β3-VEGFR2 interactions, and were stimulated with 30 ng/ml VEGF at 37°C over a time course. ECs were lysed and analysed by Western blot (WB) for protein levels of phosphorylated (phospho) and total p130cas and FAK. HSC-70 served as a loading control. Data are representative of 3 independent experiments.

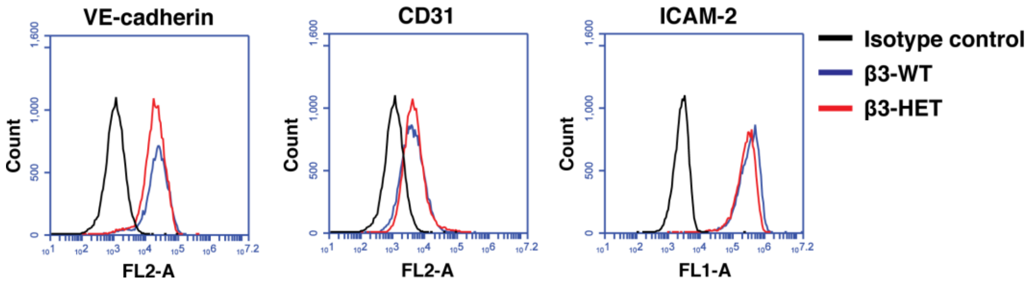

Supplementary Figure 3: Polyoma-middle-T-antigen immortialised endothelial cells maintain the endothelial identity. ECs were trypsinised and analysed by flow cytometry for surface levels of the EC markers VE-Cadherin, CD31, and ICAM-2. Median fluorescence intensity was measured after forward versus side scatter data were tightly gated around, and normalised to, an isotype control. Representative flow-cytometric histogram profiles of β3-WT and β3-HET ECs are shown.
